# Navigating the unstructured by evaluating alphafold’s efficacy in predicting missing residues and structural disorder in proteins

**DOI:** 10.1371/journal.pone.0313812

**Published:** 2025-03-25

**Authors:** Sen Zheng

**Affiliations:** Bio-Electron Microscopy Facility, iHuman Institution, ShanghaiTech University, Shanghai, China; AlloTec Bio, UNITED STATES OF AMERICA

## Abstract

The study investigated regions with undefined structures, known as “missing” segments in X-ray crystallography and cryo-electron microscopy (Cryo-EM) data, by assessing their predicted structural confidence and disorder scores. Utilizing a comprehensive dataset from the Protein Data Bank (PDB), residues were categorized as “modeled”, “hard missing” and “soft missing” based on their visibility in structural datasets. Key features were determined, including a confidence score predicted local distance difference test (pLDDT) from AlphaFold2, an advanced structural prediction tool, and a disorder score from IUPred, a traditional disorder prediction method. To enhance prediction performance for unstructured residues, we employed a Long Short-Term Memory (LSTM) model, integrating both scores with amino acid sequences. Notable patterns such as composition, region lengths and prediction scores were observed in unstructured residues and regions identified through structural experiments over our studied period. Our findings also indicate that “hard missing” residues often align with low confidence scores, whereas “soft missing” residues exhibit dynamic behavior that can complicate predictions. The incorporation of pLDDT, IUPred scores, and sequence data into the LSTM model has improved the differentiation between structured and unstructured residues, particularly for shorter unstructured regions. This research elucidates the relationship between established computational predictions and experimental structural data, enhancing our ability to target structurally significant areas for research and guiding experimental designs toward functionally relevant regions.

## Introduction

Proteins are fundamental to life, and understanding their three-dimensional (3D) structures is crucial for elucidating their functions. Structural biology has made significant strides, underscoring the central dogma that a protein's function is intricately linked to its 3D structure [1,2]. These advancements are reflected in resources such as the Protein Data Bank (PDB), which archives extensive structural data essential for research [3].

Three primary experimental approaches are foundational in determining protein structures: X-ray crystallography (X-ray), nuclear magnetic resonance (NMR) spectroscopy, and cryogenic electron microscopy (cryo-EM). X-ray crystallography requires proteins to be crystallized, which can be challenging for flexible regions and complex assemblies [2,4]. NMR spectroscopy provides insights into molecular flexibility but is primarily limited to smaller proteins [5]. Cryo-EM has revolutionized the field by enabling 3D reconstructions of proteins in near-native states without the need for crystallization [6–8], positioning it as a prime candidate for atomic-level resolution in the future [9]. As a developing tool, cryo-EM includes modalities such as single-particle analysis (SPA) and cryo-electron tomography, the latter improved by sub-tomogram averaging for enhanced resolution, facilitating diverse strategies for structural exploration [10,11]. Structural biology now offers a sophisticated array of techniques that allow researchers to tailor their approaches based on protein characteristics such as size, complexity, and function, as well as specific experimental objectives and conditions.

However, many proteins have regions that are not strictly structured, known as disordered regions [12]. This partially or fully “unstructured” nature can arise for various reasons, such as inconsistencies in X-ray scattering patterns [13,14] or the challenges of reconstructing precise models from fragmented density maps in cryo-EM [15,16], combined with the intrinsic dynamic nature of proteins [17]. This dynamic behavior is attributable to the fluctuating and heterogeneous conformations seen across different states, emphasizing fast transitions and flexibility [18]. Proteins marked by this variability and absence of a defined structure are called intrinsically disordered proteins (IDPs) and intrinsically disordered protein regions (IDPRs) [19]. Their flexibility allows them to traverse a spectrum of conformational states, from highly disordered random coils to pre-molten globules and molten globules [20,21], enhancing their versatility and effectiveness in functional roles such as effectors, assemblers, display sites, and scavengers [13,19,22,23]. The structural versatility of IDPs/IDPRs, while challenging to characterize by conventional structural methods, contributes significantly to biological activity and adaptability [24–26].

Classifying and predicting protein disorder is therefore a race against time and resources, given the limited number of experimentally determined structures and the vast, under-reviewed protein sequence data available in the expanding UniProt database [27]. Over a hundred traditional disorder predictors have utilized various bioinformatic approaches to help understand protein flexibility in the absence of comprehensive structural data [28]. Among these tools, IUPred assesses interaction energies within amino acid chains and has proven effective for identifying proteins and regions less likely to form stable interactions [29,30]. It remains a robust complement to sequence-based methods, maintaining its status as a reliable comparator among disorder predictors [31].

In 2022, protein structure prediction underwent a transformation with the introduction of AlphaFold2, a deep learning-based approach [32]. While it achieved breakthrough accuracy, AlphaFold2 has limitations, particularly in predicting disordered regions and loops [33]. Although the models have been consistent with experimental data, such as their successful application in resolving the N-terminal region of the PTX3 complex with cryo-EM [34], challenges persist, particularly with long loops and membrane proteins [35]. Notably, its lower accuracy in disordered regions can aid the identification of disorder when analyzed correctly. AlphaFold2’s predicted Local Distance Difference Test (pLDDT) scores have been explored for their utility in pinpointing disordered areas, sparking interest in their potential to accurately identify IDPs and IDPRs [35–38].

To enhance our understanding of protein structure representation, we developed a framework to evaluate computational predictions against experimental results. We curated a dataset of proteins featuring chains with the highest degree of completeness recorded in the PDB, categorizing residues into “modeled”, “hard missing” and “soft missing” classes. Our investigation revealed that “hard missing” residues often occur in low-confidence regions, with correlations varying by experimental method, region length, and residue type. Leveraging pLDDT scores and machine learning, we identified key features distinguishing “modeled” from “hard missing” residues, particularly with SPA from cryo-EM. Our findings underscore how complementary approaches can bridge gaps in structural prediction, especially for dynamic and unstructured protein regions, offering a sound basis for guiding more targeted structural experiments.

## Materials and methods

### Dataset

Given the diverse protein functions and categorizations of disorder [13,39], selecting a well-accepted definition to represent disorder that aligns with experimental and historical structural data is crucial. To simplify this complexity and achieve our objectives, we adopted the basic definition of disorder as “missing coordinates”, especially within the longest structural representations. Missing coordinates are a direct and observable feature in experimentally determined protein structures, providing a straightforward basis for further analysis [39,40].

Our research began with the selection of the longest complete structures from the PDB center, based on 100% sequence identity up to March 2024. We prioritized these entries due to their comprehensive nature, which limits uncertainties associated with less complete models. The absence of atomic coordinates in these structures typically flags regions that are difficult to capture, possibly due to intrinsic disorder, flexibility, or heterogeneity. Our study probes these elusive regions, assessing their characteristics using both the innovative AlphaFold2 and the traditional IUPred disorder predictors. Our objective is to decipher the inherent challenges in structurally characterizing these segments, utilizing a broad and historical collection of PDB data. This initiative is an initial step in unraveling the correlation between unstructured model areas and disorder-related attributes.

Protein entries were further grouped by UniProt accession number and sequence, and categorized into three experimental method groups: X-ray, SPA, and Tomography (Tomo), which encompasses both tomography and sub-tomogram averaging. Specifically, tomography produces low to intermediate-resolution density maps, whereas sub-tomogram averaging improves resolution by combining multiple datasets [41–43]. Given their similar principles in data collection, 3D reconstruction, and the use of deep-learning for structural interpretation, both methods were analyzed together to reflect their collective advancements in the field [44–46].

AlphaFold prediction results were sourced from AlphaFoldDB (https://alphafold.com/), and additional protein details, such as full-length sequences and secondary structures, were gathered from UniProt (https://www.uniprot.org/). Ultimately, this procedure yielded collections of 34,568, 11,072, and 416 unique protein entries for the X-ray, SPA, and Tomo datasets, respectively, with more details provided in Table 1 and S1-S3 Tables in S1 File.

**Table 1:** Basic information of dataset collected from various structural experiments in this study.

		X-ray		SPA		Tomo	
		Pre-2022	Post-2022	Pre-2022	Post-2022	Pre-2022	Post-2022
**Proteins**		31,622	2,947	4,834	6,239	142	275
**PDB entries**		55,089	3,541	6,493	6,938	166	291
**Modeled**		8,546,651	815,530	1,329,389	1,678,717	45,088	79,060
**Hard Missing**	*Short*	301,669	28,613	70,030	84,703	1,477	3,509
	*Long*	193,727	22,144	307,379	413,112	7,490	20,161
**Soft Missing**		97,843	3,430	54,457	24,148	2588	819
**Resolution**		2.2 ± 0.6		3.9 ± 2.3		14.7 ± 10.2	

### Residue and region classifications

To further determine which residues are fully or partially missing, we analyzed multiple PDB entries corresponding to the same protein and examined the presence of “Cα” carbon coordinates for each residue position in datasets. Residues were categorized as follows:

Modeled: Residues for which the “Cα” is present in all relevant PDB entries for the same residue position.
Hard Missing: Residues for which the “Cα” is absent in all related PDB entries.
Soft Missing: Residues for which the “Cα” is sometimes absent in the related PDB entries.

This classification leverages the variability in residue representation across multiple PDB entries for the same protein. For example, “soft missing” residues are identified based on partial absence by observing inconsistencies in “Cα” presence across PDB entries. The basic information related to the dataset collected from this study is summarized and listed in Table 1.

For region classification, we examined the continuum of “hard missing” residues and defined them based on length: regions with 30 or fewer residues were categorized as “short” disorder regions; those with more than 30 residues were termed “long” disorder regions. This 30-residue threshold was selected based on prior length-dependent disorder analyses [47], aiming to uncover potential correlations in predictability and distinct characteristics between shorter (≤30 residues) and longer (>30 residues) disordered segments.

### Feature extraction and correlation analysis

Key feature extraction began with retrieving pLDDT scores for protein sequences from AlphaFoldDB structure files. Disorder predictions were performed using locally installed IUPred3 software, applying the “long” method under default settings [30]. Sequences shorter than 16 amino acids, which IUPred3 could not process, were given an arbitrary score of 0 to denote their negligible disorder content. This approach assumes that such short IDRs potentially exhibit lower disorder content relative to longer ones [48]. This approach should minimize potential analytical bias given the rarity of such proteins in the whole dataset. Residues were analyzed for other features such as compositions from PDB files and secondary structure information from UniProt database.

Residue categorization, including “modeled”, “hard missing”, and “soft missing”, facilitated the examination of correlations between protein features. Pearson’s correlation was employed to compare residue type counts against individual feature counts.

For a more detailed assessment, quadrant analysis was performed by categorizing the residues into four distinct groups based on their pLDDT and IUPred scores. Thresholds were set at 70 for pLDDT and 0.5 for IUPred, based on reference suggested. Confidence scores of ≥  70 are frequently used in AlphaFold2 to identify highly reliable regions with positional conservation [49]. For IUPred, the default threshold of 0.5 is used as cutoff for distinguishing ordered and disordered regions in proteins [30].

Quadrant 1 (Q1), contains residues with high pLDDT confidence ( ≥ 70) and low IUPred disorder ( < 0.5);
Quadrant 2 (Q2), contains residues with high pLDDT confidence ( ≥ 70) but high IUPred disorder ( ≥ 0.5);
Quadrant 3 (Q3), contains residues with low pLDDT confidence ( < 70) and low IUPred disorder ( < 0.5);
Quadrant 4 (Q4), contains residues with low pLDDT confidence ( < 70) and high IUPred disorder ( ≥ 0.5).

### Prediction model preparation and training

In an attempt to understand variance in time series across different types of experiments in recent prediction tool development, protein entries from the X-ray, SPA, and Tomo groups were divided into two sets. Proteins with collected PDBs released before 2022 were assigned to the training set, while those with PDBs released after 2022 were used for validation.

For the basic pLDDT model, following established guidelines [36,40] and observations from feature analysis, residues scoring under 70 in pLDDT were assumed to be labeled as “hard missing”, and the rest as “modeled”. In the basic IUPRED model, residues with scores above 0.5 were assumed to be classified as “hard missing”, with others marked as “modeled”. The “soft missing” category was not used in these basic models due to the difficulty in detecting correlations within the dataset as observed.

To prepare for the training and further validation of an LSTM model, a foundational setup was carried out with modifications tailored to our specific needs [50]. The protein sequences were converted into tokens using a 21-dimensional representation, with an additional two dimensions for the pLDDT and IUPRED scores. Following the input layer, a LSTM layer was introduced, leading to a dense layer with a soft-max activation function. The classification targets for residues were defined under three categories: “modeled”, “hard missing” or “soft missing”, which were then used by the final classification layer. We employed the “Adam” optimizer and used the “categorical_cross-entropy” loss function. For the LSTM model, sequence lengths were standardized to 1,500 for the X-ray group with 256 neurons, and to 2,500 with 300 neurons for the SPA group, an approach that provided optimal outcomes. Additionally, we discarded entries with fewer than five “hard missing” residues to prevent a bias towards the “modeled” category, thereby increasing model validity. This step also addressed the issue that short disorder regions are often not well-represented in prediction model training [47].

Finally, two LSTM models were specifically trained for the X-ray and SPA datasets. Due to limited data, the Tomo LSTM model was not developed; the SPA LSTM model was instead validated against the Tomo dataset.

### Validation of model prediction

To assess residue classification performance, we employed precision, recall, and F1 score for the pLDDT, IUPred, and LSTM models using our validation set [50,51]. Definitions are as follows:

True Positive (TP): Correct prediction of a residue as belonging to its group.
True Negative (TN): Correct identification of a residue as not part of a specific group.
False Positive (FP): Incorrectly predicting a residue as part of a specific group when it is not.
False Negative (FN): Incorrectly predicting a residue as not part of a specific group when it is.

For instance, evaluating the “modeled” group: TP occurs when both prediction and label are “modeled”; TN when both refute “modeled”; FP if the prediction wrongly indicates “modeled”; and FN if “modeled” is missed in the prediction. These clear definitions aid accurate model assessment. The formulas for Precision, Recall, and F1 Score are:


$$Precision=TP/\left(TP+FP\right)$$

$$Recall=TP/\left(TP+FN\right)$$

$$F1Score=2\times Precision\times Recall/\left(Precision+Recall\right)$$

We further analyzed “hard missing” residues within “short” ( ≤ 30 residues) and “long” ( > 30 residues) regions, employing TP, TN, FP, and FN per residue:

TP for “short”: Correctly predicted a “short” residue as in a “short” region.
TN for “short”: Correctly predicted a residue not “short” as outside a “short” region.
FP for “short”: Incorrectly predicted a residue not “short” as in a “short” region.
FN for “short”: Incorrectly predicted a “short” residue as not in a “short” region.

Similar criteria were applied to residues in “long” regions. Residue distribution with related scores in “short” and “long” regions across structural groups is detailed in the S4-6 Tables in S1 Table, showing means and standard deviations for TP, TN, FP, and FN, facilitating understanding of prediction performance and error analysis with varied features.

### Analysis of human dataset

In an effort to practically identify those with regions likely to be structured yet unrecorded on PDB, we evaluated the performance of our classification models by analyzing human protein sequences based on their pLDDT and LSTM prediction results, supplemented with functional insights from UniProt. Proteins were grouped into two categories based on the availability of their structures according to the following rules:

1. Unsolved Sequence: This category includes entries with PDB structures partially determined, as annotated in UniProt. Specifically, protein sequence regions annotated in UniProt and linked with PDB entries were extracted. Residues annotated by UniProt as covered by PDB structures were labeled as “solved”, while all other residues were designated as “unsolved”. For instance, if a protein (e.g., A6NED2) is 376 residues long and UniProt reports PDB coverage for residues 139-143 (PDB:6F4R, 6F4S, 6FST), residues 139-143 are treated as “solved”, and residues 1-138 and 144-376 as “unsolved”. This category comprises 8,099 entries as of March 2024.
2. Undetermined Sequence: Entries lacking any associated PDB structural data fall into this category. The entire sequence of these proteins was classified as “unstructured”. As of March 2024, this category includes 11,665 entries.

To further evaluate the likelihood of residues being “modeled” and to identify high-confidence regions in uncharacterized sequences, we employed two complementary metrics:

1. pLDDT Score Distribution: Residues with pLDDT scores ≥  70 were considered to have high confidence in their structural predictions, reflecting the reliability of AlphaFold2’s predictions. The proportion of high-confidence residues (pLDDT ≥  70) within a sequence was calculated.
2. SPA-Trained LSTM Model Distribution: We also used predictions by a trained SPA-LSTM model to determine the likelihood of a residue being suitable for structural modeling. The ratio of “modeled” residues was computed within sequences.

These metrics collectively provide a robust method for assessing which sequences or regions may be “modeled” in SPA experiments, with a focus on regions displaying higher ratios of pLDDT scores ≥  70 and those predicted as “modeled” by the SPA-trained LSTM model. These sequences are considered high-priority candidates within the dataset of human proteins that have not yet been structurally resolved. The data been supplemented with functional annotations from UniProt and are mentioned in the data availability statement.

### Software

The main scripts used in this study, including data preparation and feature extraction, were written in Python (3.10.14). Pearson correlation coefficients were calculated using the pandas (2.2.1) package. LSTM models were trained using the TensorFlow (2.16.1), Keras (3.3.3), scikit-learn (1.5.0) and NumPy (1.26.4) packages. Visualization of the results, including violin and heatmap plots, was accomplished with Matplotlib (3.8.4) and seaborn (0.12.2). Codes, models and related datasets collected in this study were uploaded to https://github.com/thsformygod/Nav-pLDDT-IUPred/.

## Results

### Comparative assessment of structural completeness across experimental methods

To investigate the distribution of unstructured residues and related features, we collected a diverse dataset of protein structures from the PDB. Multiple PDBs for the same protein were grouped and further categorized based on structural experimental methods. This dataset, summarized in Table 1, serves as the foundation for our study.

This dataset reveals that the most complete structures are historically dominated by X-ray crystallography. Additionally, SPA has contributed notably to the collection of intact structures, especially post 2022. The Tomo group demonstrated a smaller footprint but has also shown a quickly rising trend recently. Notably, X-ray crystallography has historically contributed to high-resolution structure determination, followed by the also high-resolution contributions of SPA and more intermediate resolutions in the Tomo group.

To assess unstructured residues, we analyzed missing coordinates in the longest chains of these proteins, representing both intact structures and structurally challenging regions with disorder. Missing residues were classified as “hard missing” (consistently absent across chains for protein) or “soft missing” (variably absent). Specifically, our revised data show that X-ray structures had more instances of short “hard missing” residues than long “hard missing” residues. For both SPA and Tomo groups, fewer short “hard missing” residues compared to long “hard missing” residues were noted.

This variation indicates methodological differences in achieving structural completeness. X-ray predominantly exhibited short “hard missing” residues due to crystallization challenges with longer disordered segments, while SPA and Tomo showed higher frequencies of missing residues in long unstructured regions, highlighting Cryo-EM’s ability to better accommodate extended disordered segments than X-ray.

Overall, this dataset contains valuable resource to provide a basis for examining current developments, revealing notable variability in how experimental methods handle structural disorder.

### Correlation of features with residue groups using pearson analysis

In this section, we analyzed the distribution of “modeled” and “missing” residues to uncover key differences across experimental techniques. Residue counts were correlated with specific features for each dataset using Pearson correlation tests to examine distinctions among “modeled”, “hard missing”, and “soft missing” residue groups.

**Fig 1:**
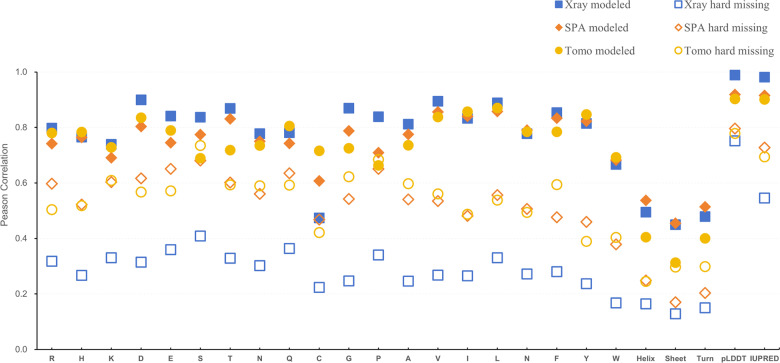
Correlation analysis of “modeled” and “hard missing” with structural features in X-ray, SPA and Tomo experiments. In “Modeled” residues (solid), the correlations observed with high pLDDT scores ( ≥ 70), displaying Pearson correlations of 0.99 (X-ray), 0.92 (SPA) and 0.90 (Tomo), and with ordered IUPred scores ( < 0.5), showing correlations of 0.98 (X-ray), 0.92 (SPA), and 0.90 (Tomo). The next correlation groups are amino acid residue types, then followed by the residue counts of secondary structure types. In “Hard missing” residues (hollow), highest correlation is found with low pLDDT scores ( < 70) and disordered IUPred scores ( > 0.5), with correlations of 0.75 and 0.55 for X-ray, 0.80 and 0.73 for SPA, and 0.78 and 0.69 for Tomo. The amino acid residue types and counts of secondary structure types show slight correlations. The “Soft missing” residue (not showed) group show minimum correlations from 0.1-0.3 for all these features. X-ray, square; SPA, diamond; Tomo, circle.

As shown in Fig 1, for “modeled” residues, high pLDDT scores ( ≥ 70) emerged as the most indicative feature, with strong correlations of 0.99, 0.92, and 0.90 for X-ray, SPA, and Tomo, respectively. Similarly, residues with ordered IUPred scores ( < 0.5) showed strong correlations of 0.98, 0.92, and 0.90 across these methods.

In contrast, “hard missing” residues were strongly linked to low pLDDT scores (<70) and disordered IUPred scores (≥0.5), with correlation coefficients of 0.75 and 0.55 for X-ray, 0.80 and 0.73 for SPA, and 0.78 and 0.69 for Tomo. These trends confirm the association between predictive metrics and challenges in modeling unstructured regions. However, the “soft missing” group exhibited no clear linear correlations with these scores, suggesting that its characteristic complexity is not fully captured by such single linear correlation measurements.

Examining amino acid composition further discriminated between residue groups. The “hard missing” residues were more enriched in polar or charged residues (K, E, S, P) while being depleted in hydrophobic residues (W, Y, F, L, I, V) compared to “modeled” ones (S3 Table in S1 Table), consistent with the characteristics of IDRs [52]. Interestingly, “soft missing” residues displayed an intermediate composition between these groups, underscoring their indeterminate nature. Specifically, for the “X-ray” dataset collected here, a compositional analysis (S2 Table in S1 Table) revealed similarities to a previous reported dataset PDB-25 [53–55], while “SPA” and “Tomo” datasets were more aligned between the PDB-25 and another unbiased Swiss-Prot dataset in most residue types.

Notably, counts of residues forming secondary structure elements (such as helices, sheets, and turns) did not exhibit correlations comparably to other features, suggesting that measuring counts of secondary structure type alone is insufficient for predicting structural presence. Previous studies have shown that using secondary structure elements for disorder prediction often fails due to the lack of residue-level resolution in detection methods [51]. This aligns with our findings, as simple linear correlations may not capture the complex interplay of secondary structures in transitions of these unstructured regions [56].

Overall, this correlation analysis highlights pLDDT and IUPred scores as valuable predictive features for differentiating “modeled” from “hard missing” residues. Amino acid composition also plays a critical role for indicating unstructured ones, while the complexity of “soft missing” residues remains challenging to characterize with single features we tested so far.

### Insights into the landscape of pLDDT and IUPred scores across residue groups and experiments

Based on the correlation analysis that found key features, pLDDT and IUPred scores were investigated by their distribution in different groups across X-ray, SPA, and Tomo datasets, to understand how these scores reflect prediction of structural reliability and disorder, providing overview for classifying “modeled” versus “missing” residues.

Residues were categorized into four quadrants based on pLDDT and IUPred scores: Q1 consists of residues with high pLDDT confidence and IUPred order; Q2 includes residues with high pLDDT confidence but IUPred disorder; Q3 comprises residues with low pLDDT confidence but IUPred order; and Q4 contains residues with low pLDDT confidence and IUPred disorder.

**Fig 2:**
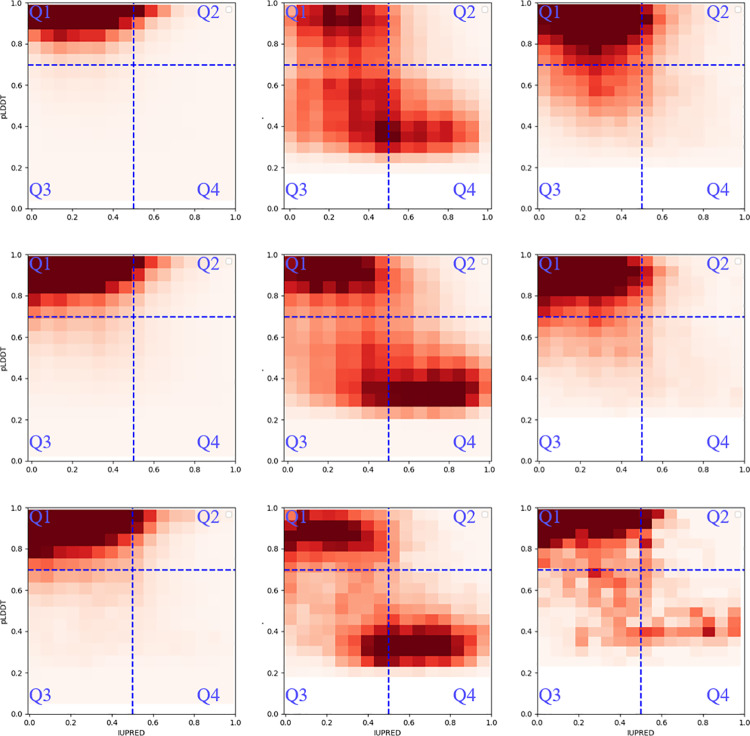
Distribution of residue categories across quartiles in X-ray, SPA, and Tomo Data. The distribution of “Modeled” (Left), “Hard missing” (Middle) and “Soft missing” (Right) residue group split with Q1 to Q4 across X-ray (Up row), SPA (Middle row), and Tomo (Down row) are represented as a density heatmap. Darker color corresponds for higher condensed population.

Fig 2 and S1 Table in S1 Table summarize these findings. In X-ray data, “modeled” residues predominantly reside in Q1 (93.6%) with a minor presence in Q2. “Hard missing” residues are more evenly distributed to low confidence area, particularly in Q3, indicating structural uncertainty. “Soft missing” residues mostly appear in Q1 (63.7%), with some distribution in Q3 (20.1%).

In SPA data, “modeled” residues cluster in Q1 (86.6%) with slight increases in Q2 and Q3 compared to X-ray data. “Hard missing” residues demonstrate a similar distribution to X-ray, spread across Q1, Q3, and Q4, albeit with varying intensity. “Soft missing” residues are primarily in Q1 (73.2%), with a reduced presence in Q3 and minimal representation in Q2 and Q4.

For Tomo data, “modeled” residues mainly occupy Q1 (84.2%), consistent with X-ray and SPA trends. “Hard missing” residues are distributed across Q1, Q3, and Q4, with a slightly reduced concentration in Q3. “Soft missing” residues are largely found in Q1 (65.3%), with notable representation in Q3 and increased presence in Q4 compared to X-ray and SPA datasets.

Overall, in “modeled” group, Q1 consistently houses the majority of residues in all methods, indicating that high pLDDT and low IUPred scores are reliable markers of structurally stable and ordered regions (S1 Table in S1 Table). The distribution patterns’ similarity across different methods suggests that these techniques correspondingly reflect predicted confidence and disorder scores.

The “hard missing” group shows more variability, particularly in Q3 and Q4, where lower pLDDT scores indicate uncertainty in structural predictions (S1 Table in S1 Table). The distribution across Q1, Q3, and Q4 underscores the challenges in predicting these residues. Notably, there is a higher occupancy in Q3 as compared to Q2, suggesting a potential underlying relationship between these two distinct prediction factors that merits further investigation.

“Soft missing” residues demonstrate intermediate patterns between the other two groups, clustered predominantly in Q1 but also appearing in Q3 across all methods (S1 Table in S1 Table). The higher presence of “soft missing” residues in Q4 within the Tomo data suggests a greater disorder and low confidence in structural predictions compared to X-ray and SPA, which may be due to the different techniques and generally lower resolution of reconstructed structures in tomography.

These findings highlight the distinct characteristics of pLDDT and IUPred scores for “modeled” versus “missing” residues, illustrating how these scores are distributed among partially or fully structured residues and those found in inherently disordered or ambiguous regions.

### Predictive modeling of residue groups post 2022

This section investigates the correlation between pLDDT and IUPred scores with missing coordinates across various structural experiments, particularly after the release of prediction algorithms. We evaluated the predictive capabilities of two fundamental models (pLDDT and IUPred) alongside an advanced LSTM model, utilizing data collected post 2022. The LSTM model excels in capturing features in long-range sequential data through memory blocks [57], allowing for effective classification of residues into “modeled”, “hard missing” and the more intermediate “soft missing” categories.

To classify residues, we hypothesized, based on prior analyses and references, that residues with pLDDT scores below 70 or IUPred scores above 0.5 are categorized as “hard missing”, while the others fall into the “modeled” category [36,40]. However, the “soft missing” group was not assigned specific pLDDT or IUPred scores at this stage, as indicated by previous heatmaps and weaker correlations in this dataset.

To classify residues including “soft missing” group, we trained the LSTM model and incorporated sequence context. The dataset was split into a training set (entries before 2022) and a validation set (post 2022 entries). The LSTM was trained on sequences with accompanying pLDDT and IUPred scores, and its performance was subsequently validated on the latest data.

Performance comparisons among these models for residue groups were assessed using precision, recall, and F1 scores, as summarized in Table 2. For “modeled” residues, the LSTM model demonstrated exceptional performance, achieving F1-scores of 0.98 (X-ray), 0.91 (SPA), and 0.91 (Tomo), closely followed by the pLDDT model. The IUPred model, in contrast, performed least well.

**Table 2:** Summary of residue groups predicted by different methods.

	Method	X-ray			SPA			Tomo		
		Precision	Recall	F1score	Precision	Recall	F1score	Precision	Recall	F1score
**Modeled**	pLDDT	0.97	0.96	0.97	0.89	0.92	0.90	0.89	0.90	0.89
	IUPred	0.95	0.96	0.95	0.83	0.93	0.88	0.83	0.92	0.87
	LSTM	0.97	0.98	0.98	0.88	0.94	0.91	0.88	0.93	0.91
**Hard Missing**	pLDDT	0.53	0.65	0.58	0.69	0.64	0.66	0.64	0.65	0.64
	IUPred	0.27	0.30	0.28	0.63	0.43	0.51	0.57	0.41	0.47
	LSTM	0.67	0.51	0.58	0.75	0.59	0.66	0.70	0.60	0.65

In predicting “hard missing” residues, the LSTM maintained its lead, achieving F1-scores of 0.58 (X-ray) and 0.66 (SPA), slightly surpassing the pLDDT model for Tomo with scores of 0.65 versus 0.64. However, for “soft missing” residues, the LSTM did not classify any during validation, likely due to low correlation efficiency and a scarcity of data points of this class.

Further, we evaluated the prediction performance for “hard missing” regions by distinguishing “short” ( ≤ 30 contiguous “hard missing” residues) and “long” ( > 30 contiguous “hard missing” residues) categories, as summarized in Table 3. Results indicated that the LSTM’s F1-scores for “short” regions were 0.52/0.43/0.36 (X-ray/SPA/Tomo), outperforming the pLDDT’s scores of 0.51/0.40/0.34. Conversely, for “long” regions, the pLDDT model slightly edged ahead with F1-scores of 0.49/0.62/0.65 over the LSTM’s scores of 0.44/0.61/0.65. This reflects a trend seen in previous studies, which noted that predictors generally exhibit lower accuracy for “short” disorder regions, possibly due to their different composition and location on proteins [47,48]. Additionally, we observed a decrease in F1-scores for residues in both “short” and “long” regions compared to the overall ‘hard missing’ residues, which can be primarily attributed to an increased number of false positives (FPs) when taking into account the length of the regions as an additional factor in measuring performance.

**Table 3:** Summary of “Hard missing” regions predicted by different methods.

	Method	X-ray			SPA			Tomo		
		Precision	Recall	F1score	Precision	Recall	F1score	Precision	Recall	F1score
**Short**	pLDDT	0.42	0.66	0.51	0.30	0.62	0.40	0.24	0.57	0.34
	IUPred	0.15	0.24	0.19	0.14	0.23	0.17	0.07	0.14	0.09
	LSTM	0.55	0.5	0.52	0.35	0.57	0.43	0.28	0.50	0.36
**Long**	pLDDT	0.54	0.45	0.49	0.75	0.53	0.62	0.74	0.58	0.65
	IUPred	0.41	0.21	0.28	0.73	0.35	0.47	0.67	0.33	0.44
	LSTM	0.60	0.34	0.44	0.79	0.50	0.61	0.78	0.55	0.65

A detailed analysis revealed significant prediction patterns, particularly regarding the disparities in “hard missing” residues between pLDDT, IUPred, and LSTM models (S4-S6 Tables in S1 Table). While true positive residues remained consistent across models, IUPred showed increased variance in false positives and false negatives. Notably, residues from “short” regions exhibited more structured characteristics, as indicated by higher pLDDT scores and more orderly scores from IUPred, in contrast to those from “long” regions which exhibited the opposite trend, particularly in the SPA dataset. These observations may elucidate prediction discrepancies between “short” and “long” regions across different models and experiments.

Both the basic pLDDT approach and the LSTM model demonstrated effectiveness in classifying “hard missing” residues, highlighting their ability to identify likely unstructured regions. The neural network model shows potential for further improvement, possibly with the refinement of training methodologies [58]. With appropriate optimizations, it has the potential to become a more fine-tuned tool, promising performance advancements in tasks such as identifying unstructured regions. Meanwhile, the use of pLDDTs in predicting extended segments of protein structures could serve as a dependable baseline method in most cases.

### Application of pLDDT and LSTM models in analyzing human protein dataset

**Fig 3:**
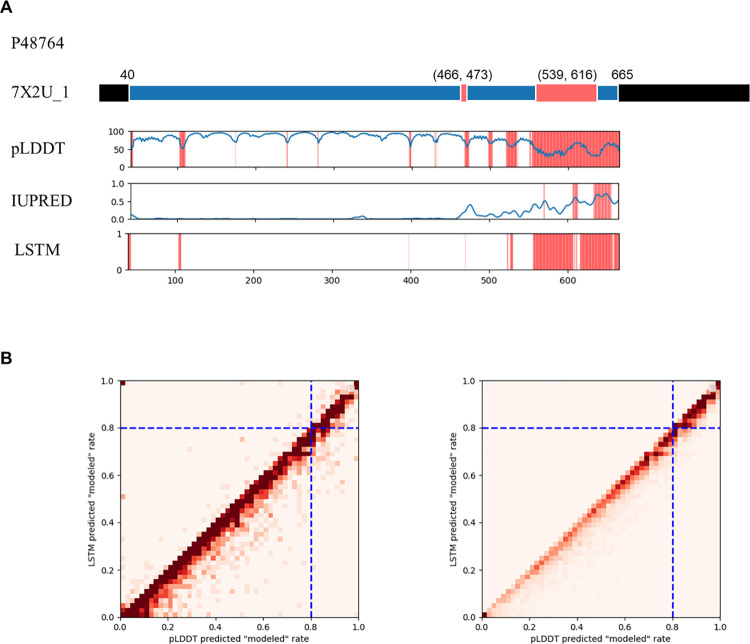
Example of prediction results and predicted “modeled” residue of human protein. (A) The full-length example of the protein SLC9A3 with structure solved (7X2U_1) and aligned with prediction results of models is depicted, as “modeled” (blue) segments, “hard missing” (red) and rest of unmodeled segments (black). (B) The density heatmap plots the predicted “modeled” rates from the pLDDT and LSTM models, on human protein dataset. The blue dashed lines indicate thresholds for high confidence in “modeled” (≥80%) predictions, including 322 “unsolved sequence” entries (left) and 3179 “undetermined sequence” entries exceeding 200 residues (right).

The human protein SLC9A3 (Sodium/hydrogen exchanger 3, UniProt P48764) serves as an example to illustrate the efficacy of distinct models in classifying “modeled” and “hard missing” residues. Fig 3A shows the protein structure spanning residues 40 to 665 (PDB: 7X2U, released on 2022-04-20, reconstructed using SPA), with “hard missing” segments between positions 466-473 (“short”) and 539-616 (“long”), demonstrating a general concordance between predicted and experimental structural data. Notably, both models marked the region 616-665 as potentially unstructured possibly due to its functional role in interactions. This aligns with UniProt annotations, supporting its key functional significance (e.g., residues 590-667 interaction with NHERF4; 591-695 interaction with AHCYL1). Therefore, using findings from this study, aligned with large knowledgebase with rich resources such as UniProt, we can more easily and precisely identify unstructured but functionally significant regions, providing valuable candidates for further investigation.

Expanding on this application, our study focuses on unsolved human proteins with the aim of identifying those with regions likely to be structured yet unrecorded. We divided the human entries into two groups: “unsolved sequence”, comprising 8,099 entries with partially solved structures, and “undetermined sequence”, encompassing 11,665 entries with no PDB coverage as of March 2024. The objective is to identify residues and regions within these sequences with a likelihood of being “modeled”, thus aiding future structural exploration. By employing the basic pLDDT model in conjunction with the LSTM model trained for this purpose, we evaluated regions that have not been experimentally explored structurally. We prioritized those predicted to have a high “modeled” ratio, as detailed in Fig 3B. The comparison revealed a strong correlation in the “modeled” rates predicted by both pLDDT and LSTM, indicating that the LSTM model refines pLDDT predictions for unstructured residues and agrees with those for structured residues.

Through this analysis, our findings identified 3179 “undetermined sequence” entries with a high probability of containing “modeled” residues (≥80% prediction ratio by both models, involving over 200 residues), and 322 “unsolved sequence” entries similarly indicative of abundant potential “modeled” residues.

In differentiating “hard missing” residues, the choice between the basic pLDDT and the LSTM model hinges on specific research objectives: the LSTM model is more adept at identifying short unstructured regions and demonstrates better performance in precision and F1-scores, while the pLDDT model is the preferred option for analyzing longer sequences. For broader applications, such as proteome screening and disorder pre-screening, IUPred offers a rapid and accessible option for assessing protein disorder, utilizing a fundamentally distinct methodology from that of structural predictions [31]. Furthermore, incorporating functional insights from databases such as UniProt can further refine the interpretation of structurally predicted regions, thereby fostering a more integrated approach between structural and functional studies.

## Discussion

Disordered regions in proteins, although occasionally absent from structural maps, play crucial roles in protein function and present significant challenges in structural determination. Recent advancements in structure prediction, particularly with AlphaFold2, have revealed a promising correlation between low-confidence regions and intrinsic disorder across diverse species [33,38,59,60]. Data from AlphaFoldDB corroborates the prevalence of low pLDDT scores in IDRs, underscoring the utility of structure prediction methods in exploring the largely uncharted protein universe [61–63]. This approach provides valuable insights into disordered regions associated with human proteins, diseases, and the broader protein landscape.

Previous studies have often been limited by the availability of structural prediction datasets or relied on earlier versions of disorder databases like DisProt [40,59,64]. Our investigation aims to bridge these gaps by utilizing a comprehensive dataset that integrates both recent and historical structural data derived from various experimental techniques. This broad comparative framework allows for an in-depth exploration of the interplay between structured and unstructured residues. Among the models tested, the LSTM model demonstrated potential due to its capability to capture long-range dependencies [57]. However, all three models proposed here struggled with effectively classifying “soft missing” residues, those at the boundary between structured and unstructured regions, highlighting the complexities inherent in this task.

This study not only evaluates prediction tools like AlphaFold2 and IUPred but also seeks to provide a comprehensive understanding of structural disorder across experimental datasets. By classifying residues into “modeled”, “hard missing” and “soft missing” categories, we elucidate the intricate relationships between types of missing residues, their regions, and the distribution of prediction scores. “Modeled” residues generally achieve high pLDDT scores and are predominantly found in regions where AlphaFold2’s predictions are most reliable, particularly in data derived from X-ray crystallography, which exhibits the highest median pLDDT scores. In contrast, “hard missing” residues are largely situated within low-confidence regions, underscoring their unpredictability for structure prediction models [61,62,65,66].

The score distributions obtained from IUPred add another layer of differentiation by indicating the likely ordered or disordered regions, thus suggesting varying levels of predictability [31,51]. However, the decline in IUPred’s performance may be multifaceted; one factor could be the indiscriminate use of the “long” method across the dataset, without considering the specific context of “short” versus “long” disordered regions. Additionally, the choice of default thresholds for disorder prediction might not be optimal. A more meticulous selection of parameters, tailored to the specific focus of evaluation, could potentially enhance the explanatory power of these conventional disorder prediction tools.

“Soft missing” residues represent an intriguing boundary case in our study, poised at the dynamic intersection between order and disorder. This oscillatory nature complicates their predictability, as highlighted by the distributions of disorder-related scores place these residues near the thresholds that distinguish ordered from disordered regions [40,49].

To tackle these challenges, strategies might include addressing dataset imbalances and improving feature representation. Assigning class weights during model training could heighten sensitivity to underrepresented categories like “soft missing” residues [58], enhancing detection accuracy. Additionally, incorporating features that capture fine-grained distinctions, like solvent accessibility or incremental disorder indicators, could enrich the representation of transitional residue states, thereby improving predictive performance.

Notably, Tomo data shows a higher presence of “soft missing” residues in Q4 compared to X-ray and SPA datasets, which should be interpreted cautiously due to the smaller dataset size and the lower-to-intermediate resolution of tomography density maps [44]. However, ongoing advances in tomography techniques, faster data collection, and improved sub-tomogram averaging reconstruction processes are expected to enhance data robustness and provide clearer insights into these elusive structures in the future [41,43,45].

The discrepancy in prediction performance between “long” and “short” regions in the trained LSTM model likely reflects the length distributions of IDRs across experiments. For example, longer regions are relatively scarce in X-ray group, because they more significantly impair crystallization compared to shorter ones [2,4]. These class imbalances also pose challenges for the LSTM model, similar to those encountered in distinguishing “soft missing” residues. Future work could address these issues by applying fine-tuned class weighting during training to enhance predictions by more specifically classifying [58]. Additionally, the slight advantage observed for pLDDT in predicting “long” regions may result from the use of a deeper network architecture, which incorporates more comprehensive sequence and structural contexts [32]. Ongoing updates to LSTM training have already shown improvements, with F1-scores increasing to 0.60 (X-ray, “hard missing”) and 0.67 (SPA, “hard missing”), exceeding slightly pLDDT’s baseline performance of 0.58 and 0.66, respectively (data not shown). This suggests potential for further optimization, alongside the need for careful pre-processing and balanced dataset organization.

Our study did not find a direct link between the counts of residues within each experimentally determined secondary structure and the occurrence of missing residues. While AlphaFold2 effectively predicts secondary structures such as α-helices and β-sheets, there was also no significant correlation observed between the residue counts of these predicted secondary structures and unstructured residues (data not shown). As highlighted by CAID [51], secondary structure information alone may not suffice for accurate disorder prediction, emphasizing the complexity of unstructured regions. Although disorder is characterized by the absence of stable secondary and tertiary structures, it can still exhibit transient secondary structures necessary for function under certain conditions [56]. Experimental techniques like NMR, circular dichroism, and far-UV spectroscopy provide insights into the dynamic structural behavior of disordered regions [38,65]. Integrating these experimental approaches with computational methods holds promise for advancing our understanding of unstructured regions.

Re-evaluating features such as pLDDT distributions across experimental methods reveals structural determination challenges associated with specific techniques. For instance, X-ray crystallography excels in resolving regions with high pLDDT scores, indicating folded and stable regions. Conversely, cryo-EM mitigates crystallization challenges, making it useful under more diverse experimental conditions but with lower resolution. High pLDDT scores are generally associated with high confidence in predicted structures, whereas low pLDDT scores often indicate regions with increased flexibility and heterogeneity, presenting challenges for precise structural prediction. Additional factors such as related homologous sequences or composition bias should also be considered in these contexts [49,60,64].

In our dataset, fewer instances were observed where disorder-predicted residues had high structural prediction confidence (Q2) compared to residues predicted to be ordered, which had low prediction confidence (Q3), especially among unstructured residues. This pattern spans various structural experimental outcomes, suggesting that unstructured residues tend to correlate more consistently with low structural prediction confidence in this study. This alignment underscores the importance of continually updating disorder annotations, as highlighted by the newly released DisProt [67].

AlphaFold2 and IUPred exhibit complementary strengths, as AlphaFold2 integrates deep-learning reliability measures, providing detailed pLDDT scores that assess the confidence of structural predictions for each residue [32,35]. IUPred, on the other hand, utilizes a statistical potential-based approach to predict disorder, relying on residues’ inability to form favorable interactions due to biophysical principles [30]. While AlphaFold2 offers comprehensive sequence and structural context, IUPred provides rapid and interpretable predictions across proteomes within minutes [31]. Together, these methods offer a synergistic approach to understanding and annotating intrinsic disorder, emphasizing the importance of integrating diverse computational tools for comprehensive protein analysis.

Exclusive reliance on computational predictions carries inherent risks, as certain cases may require complementary experimental validation [49,68]. This study primarily utilized structural data from X-ray crystallography and Cryo-EM due to their comprehensive datasets, which facilitate the analysis of correlations between unstructured regions, AlphaFold2 confidence scores, and IUPred disorder predictions. However, regions lacking resolved structural models can benefit from additional experimental techniques such as NMR, mass spectrometry, electron paramagnetic resonance, small-angle X-ray scattering, and Förster resonance energy transfer. These methods provide valuable insights into the dynamics and behavior of unstructured regions [65,69–71]. Incorporating these experimental approaches with computational predictions, particularly those leveraging diverse physical principles, would offer a more holistic understanding of these elusive and flexible regions [36,72].

Proteins are inherently complex and dynamic molecules with diverse functions, making the prediction of unstructured regions particularly challenging. Relying on a single solution is often impractical, as each protein’s specificity and unique characteristics must be considered [31,36,56]. Accurate prediction requires integrating multiple approaches and continuously refining models to capture the nuances of protein dynamics and structure.

Future work would be focused on curating improved datasets, integrating advanced features, and refining predictive models to better characterize structural boundaries. Enhancing feature representation, addressing dataset imbalances, and incorporating complementary experimental data are critical steps toward more accurate and reliable predictions. Additionally, exploring the dynamic nature of “soft missing” residues and their functional implications will deepen our understanding of protein structure and function. By advancing both computational and experimental methodologies, we can continue to unravel the complexities of protein structures, fostering innovations in biology and medicine.

## Conclusions

This study demonstrates that merging computational and experimental approaches yields deeper insights into both the structured and unstructured regions of proteins. By integrating these methods, we enhance our comprehension of protein flexibility and better inform subsequent structural and functional analyses. Specifically, our approach underscores the importance of rapid evaluations based on key features derived from an extensive utilization of current databases, including PDB, UniProt and AlphafoldDB. Through the analysis of missing coordinates and unstructured regions using prediction scores and advanced modeling techniques, we have devised a comprehensive strategy that primarily targets unstructured segments. This strategy illuminates the intricate relationship between structural determination methods and prediction tools across various notable aspects and elements. Consequently, this approach offers a broader and more discerning perspective on protein flexibility, establishing a foundational basis for future structural and functional interpretations and stressing the importance of utilizing advanced prediction tools in tandem with the wealth of accessible databases available today.

## Supporting information

S1 File S1 Fig. Compositional Distribution of Residue Categories in Structural Determination Methods.
The figure displays the counts of residues classified as “Modeled” (**left**), “Hard missing” (**middle**), and “Soft missing” (**right**) across various structural experiments, including X-ray (**blue**), SPA (**orange**), and Tomo (**yellow**). The median counts of “Modeled” residues per protein are 242 for X-ray, 190 for SPA, and 179 for Tomo. For “Hard missing” residues, the median counts are 5 for X-ray, 25 for SPA, and 11 for Tomo. Median counts for “Soft missing” residues are not reported. **S2 Fig. Distribution of Residue Scores in Different Experimental Methods.** (A) The distribution of pLDDT residue scores (ranging from 0 to 100) across the “Modeled”, “Hard missing”, and “Soft missing” groups is presented for X-ray, Single-Particle Analysis (SPA), and Tomography (Tomo) experiments. In the X-ray dataset, the “Modeled” group shows the highest median pLDDT score at 97.1, followed by the “Soft missing” group at 84.4 and the “Hard missing” group at 55.5. For the SPA dataset, the medians are 92.7 for “Modeled”, 86.7 for “Soft missing”, and 54.5 for “Hard missing”. In the Tomo dataset, medians are 91.5 for “Modeled”, 88.6 for “Soft missing”, and 52.3 for “Hard missing”. B) The distribution of IUPred scores (ranging from 0 to 1, with scores over 0.5 indicating disorder) is shown for the same groups and experimental methods. In the X-ray experiment, the “Modeled” group has the lowest median IUPred score at 0.2, followed by “Soft missing” at 0.29 and “Hard missing” at 0.38. In SPA, the median IUPred scores are 0.19 for “Modeled”, 0.23 for “Soft missing”, and 0.43 for “Hard missing”. In Tomo, the scores are 0.2 for “Modeled”, 0.32 for “Soft missing”, and 0.42 for “Hard missing”. These findings highlight distinct score distributions across experimental methods and residue categories. **S1 Table.** Distribution of residues classified by types from different methods. **S2 Table.** Composition of amino acid residues for each dataset. **S3 Table.** Composition of structured and unstructured amino acids from this study. **S4 Table.** Prediction scores of “Hard missing” residues by different models in regions from X-ray dataset. **S5 Table.** Prediction scores of “Hard missing” residues by different models in regions from SPA dataset. **S6 Table.** Prediction scores of “Hard missing” residues by different models in regions from Tomo dataset.
(DOCX)
