# Supplementary material for: Navigating the unstructured by evaluating alphafold’s efficacy in predicting missing residues and structural disorder in proteins
Source: PLoS One. 2025 Mar 25;20(3):e0313812. doi: 10.1371/journal.pone.0313812 (PMC11936262; doi:10.1371/journal.pone.0313812)
Supplement: S1 File — The figure displays the counts of residues classified as “Modeled” (left), “Hard missing” (middle), and “Soft missing” (right) across various structural experiments, including X-ray (blue), SPA (orange), and Tomo (yellow). The median counts of “Modeled” residues per protein are 242 for X-ray, 190 for SPA, and 179 for Tomo. For “Hard missing” residues, the median counts are 5 for X-ray, 25 for SPA, and 11 for Tomo. Median counts for “Soft missing” residues are not reported. S2 Fig. Distribution of Residue Scores in Different Experimental Methods. (A) The distribution of pLDDT residue scores (ranging from 0 to 100) across the “Modeled”, “Hard missing”, and “Soft missing” groups is presented for X-ray, Single-Particle Analysis (SPA), and Tomography (Tomo) experiments. In the X-ray dataset, the “Modeled” group shows the highest median pLDDT score at 97.1, followed by the “Soft missing” group at 84.4 and the “Hard missing” group at 55.5. For the SPA dataset, the medians are 92.7 for “Modeled”, 86.7 for “Soft missing”, and 54.5 for “Hard missing”. In the Tomo dataset, medians are 91.5 for “Modeled”, 88.6 for “Soft missing”, and 52.3 for “Hard missing”. B) The distribution of IUPred scores (ranging from 0 to 1, with scores over 0.5 indicating disorder) is shown for the same groups and experimental methods. In the X-ray experiment, the “Modeled” group has the lowest median IUPred score at 0.2, followed by “Soft missing” at 0.29 and “Hard missing” at 0.38. In SPA, the median IUPred scores are 0.19 for “Modeled”, 0.23 for “Soft missing”, and 0.43 for “Hard missing”. In Tomo, the scores are 0.2 for “Modeled”, 0.32 for “Soft missing”, and 0.42 for “Hard missing”. These findings highlight distinct score distributions across experimental methods and residue categories. S1 Table. Distribution of residues classified by types from different methods. S2 Table. Composition of amino acid residues for each dataset. S3 Table. Composition of structured and unstructured amino acids from th [file pone.0313812.s001.docx]

# Supplement


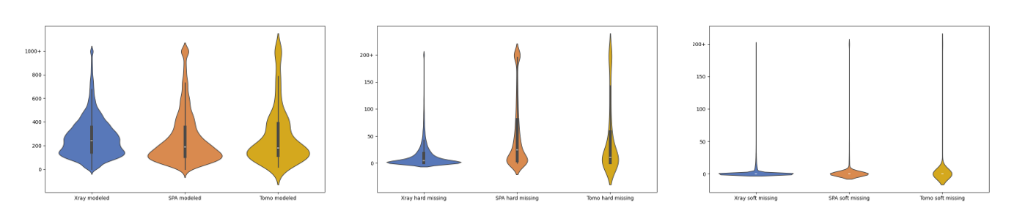


**S1 Fig.**


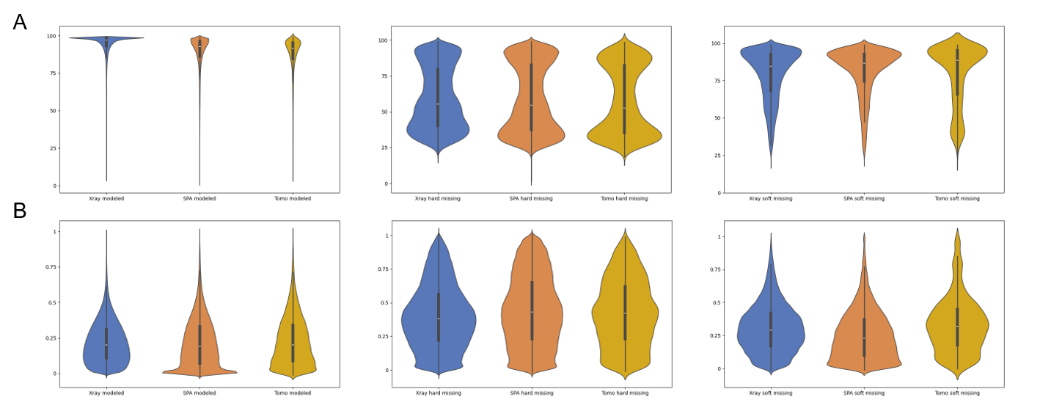


**S2 Fig.**

| **S1 Table. Percentage distribution of residues classified by types from different methods** | | | | |  |
| --- | --- | --- | --- | --- | --- |
| **Group** | **Method** | **Q1** | **Q2** | **Q3** | **Q4** |
| **Modeled** | Xray | 93.56 | 3.63 | 1.86 | 0.34 |
|  | SPA | 86.63 | 4.92 | 6.13 | 1.31 |
|  | Tomo | 84.23 | 5.69 | 7.01 | 1.93 |
| **Hard Missing** | Xray | 28.56 | 4.65 | 37.75 | 26.84 |
|  | SPA | 30.23 | 5.63 | 28.21 | 33.97 |
|  | Tomo | 33.40 | 3.17 | 27.09 | 34.23 |
| **Soft Missing** | Xray | 63.65 | 8.05 | 20.13 | 5.98 |
|  | SPA | 73.22 | 5.33 | 14.95 | 4.85 |
|  | Tomo | 65.34 | 5.46 | 15.15 | 11.89 |

1. Group: refer to residues from “modeled”, “Hard missing” and “Soft missing” found in this study
2. Method: This study analyzes PDB entries determined through various structural experiments, including X-ray crystallography (X-ray), single particle analysis (SPA), tomography and sub-tomogram averaging (Tomo).
3. Q1: Residues with pLDDT scores ≥ 70 and IUPred < 0.5.
4. Q2: Residues with pLDDT scores ≥ 70 and IUPred ≥ 0.5.
5. Q3: Residues with pLDDT scores < 70 and IUPred < 0.5.
6. Q4: Residues with pLDDT scores < 70 and IUPred ≥ 0.5.

| **S2 Table. Composition of amino acid residues for each dataset** | | | | | | |
| --- | --- | --- | --- | --- | --- | --- |
|  | **Xray** | | **SPA** | | **Tomo** | |
|  | **Before 2022** | **Behind 2022** | **Before 2022** | **Behind 2022** | **Before 2022** | **Behind 2022** |
| R | 5.2 | 5.2 | 5.9 | 5.9 | 5.3 | 5.1 |
| H | 2.3 | 2.3 | 2.3 | 2.2 | 2.2 | 2.0 |
| K | 5.8 | 5.7 | 6.6 | 6.7 | 6.7 | 7.4 |
| D | 5.9 | 5.9 | 5.2 | 5.1 | 5.5 | 4.9 |
| E | 6.9 | 6.6 | 6.8 | 6.9 | 7.0 | 6.9 |
| S | 6.1 | 6.2 | 6.8 | 6.8 | 6.9 | 7.4 |
| T | 3.7 | 3.7 | 4.1 | 4.2 | 4.3 | 4.6 |
| N | 5.4 | 5.5 | 5.3 | 5.3 | 5.7 | 5.8 |
| Q | 4.2 | 4.3 | 4.2 | 4.2 | 4.3 | 4.8 |
| C | 1.3 | 1.3 | 1.4 | 1.5 | 1.5 | 1.3 |
| G | 7.3 | 7.3 | 6.2 | 6.4 | 5.9 | 6.0 |
| P | 4.7 | 4.8 | 4.7 | 4.8 | 4.3 | 4.6 |
| A | 8.3 | 8.4 | 7.4 | 7.6 | 7.5 | 7.0 |
| V | 7.0 | 6.8 | 6.6 | 6.5 | 7.0 | 6.3 |
| I | 5.7 | 5.5 | 5.6 | 5.5 | 5.7 | 5.7 |
| L | 9.4 | 9.2 | 9.9 | 9.7 | 10.1 | 9.5 |
| M | 2.3 | 2.2 | 2.4 | 2.4 | 2.5 | 2.3 |
| F | 4.0 | 4.0 | 4.1 | 4.0 | 3.7 | 4.2 |
| Y | 3.4 | 3.5 | 3.2 | 3.2 | 3.0 | 3.1 |
| W | 1.3 | 1.4 | 1.3 | 1.2 | 1.1 | 1.0 |

| **S3 Table. Composition of structured and unstructured amino acids from this study** | | | | | | | | | |
| --- | --- | --- | --- | --- | --- | --- | --- | --- | --- |
|  | **Xray** | | | **SPA** | | | **Tomo** | | |
|  | **modeled** | **hard missing** | **soft missing** | **modeled** | **hard missing** | **soft missing** | **modeled** | **hard missing** | **soft missing** |
| R | 5.2 | 5.2 | 5.4 | 5.8 | 5.7 | 5.5 | 5.5 | 4.1 | 4.6 |
| H | 2.3 | 2.0 | 2.3 | 2.3 | 2.0 | 2.2 | 2.3 | 1.6 | 1.9 |
| K | 5.8 | 7.0 | 7.7 | 6.3 | 7.2 | 7.4 | 7.0 | 6.5 | 4.8 |
| D | 5.7 | 6.0 | 6.5 | 5.0 | 5.9 | 6.0 | 5.2 | 4.8 | 5.7 |
| E | 6.7 | 8.1 | 8.7 | 6.3 | 8.4 | 7.7 | 7.0 | 7.1 | 7.0 |
| S | 6.0 | 9.7 | 7.7 | 6.2 | 9.2 | 6.8 | 6.5 | 9.9 | 8.9 |
| T | 5.4 | 5.9 | 5.5 | 5.3 | 5.5 | 5.3 | 5.4 | 7.0 | 7.3 |
| N | 4.2 | 4.6 | 4.4 | 4.2 | 4.3 | 4.5 | 4.5 | 4.8 | 5.0 |
| Q | 3.8 | 4.6 | 4.6 | 3.9 | 4.7 | 4.4 | 4.4 | 5.1 | 4.3 |
| C | 1.5 | 1.0 | 1.3 | 1.6 | 1.2 | 1.6 | 1.5 | 0.9 | 1.4 |
| G | 7.0 | 7.9 | 7.8 | 6.2 | 6.8 | 5.8 | 5.7 | 7.6 | 6.3 |
| P | 4.6 | 6.6 | 5.5 | 4.3 | 6.3 | 4.8 | 3.9 | 6.9 | 5.1 |
| A | 8.0 | 8.8 | 7.5 | 7.3 | 8.2 | 6.7 | 7.0 | 8.1 | 9.2 |
| V | 7.1 | 4.8 | 5.3 | 7.1 | 5.2 | 6.0 | 6.8 | 5.3 | 5.7 |
| I | 5.8 | 3.2 | 3.9 | 6.2 | 3.7 | 5.5 | 6.1 | 3.8 | 4.5 |
| L | 9.6 | 6.7 | 7.4 | 10.6 | 7.8 | 9.7 | 10.3 | 7.7 | 10.1 |
| M | 2.2 | 3.3 | 2.5 | 2.4 | 2.6 | 2.2 | 2.4 | 2.6 | 2.0 |
| F | 4.1 | 2.3 | 2.8 | 4.5 | 2.7 | 3.9 | 4.1 | 3.8 | 3.3 |
| Y | 3.5 | 1.6 | 2.4 | 3.5 | 1.9 | 3.0 | 3.4 | 1.8 | 2.2 |
| W | 1.4 | 0.6 | 0.8 | 1.4 | 0.8 | 1.1 | 1.1 | 0.6 | 1.0 |

| **S4 Table. Prediction scores of “Hard missing” residues by different models in regions from X-ray dataset** | | | | | | | |
| --- | --- | --- | --- | --- | --- | --- | --- |
| **Models** |  | **pLDDT** | | **IUPred** | | **LSTM** | |
| **Regions** |  | **Short** | **Long** | **Short** | **Long** | **Short** | **Long** |
| **pLDDT Scores** | **TP** | 47.73 ± 11.23 | 39.58 ± 10.62 | 52.38 ± 18.78 | 44.37 ± 15.87 | 42.38 ± 7.98 | 36.23 ± 7.98 |
|  | **TN** | 93.32 ± 9.38 | 92.38 ± 10.65 | 92.49 ± 10.79 | 92.01 ± 11.50 | 92.89 ± 9.81 | 92.21 ± 10.95 |
|  | **FP** | 58.02 ± 9.76 | 44.61 ± 12.03 | 87.05 ± 16.75 | 78.72 ± 21.53 | 46.89 ± 7.86 | 38.88 ± 9.00 |
|  | **FN** | 77.92 ± 18.03 | 79.27 ± 17.09 | 59.89 ± 20.03 | 66.09 ± 24.40 | 73.80 ± 15.57 | 74.63 ± 19.40 |
| **IUPred Scores** | **TP** | 0.39 ± 0.23 | 0.51 ± 0.26 | 0.64 ± 0.11 | 0.75 ± 0.13 | 0.41 ± 0.24 | 0.53 ± 0.27 |
|  | **TN** | 0.23 ± 0.15 | 0.23 ± 0.15 | 0.22 ± 0.14 | 0.23 ± 0.15 | 0.23 ± 0.15 | 0.23 ± 0.15 |
|  | **FP** | 0.31 ± 0.19 | 0.33 ± 0.21 | 0.58 ± 0.08 | 0.67 ± 0.12 | 0.36 ± 0.22 | 0.33 ± 0.22 |
|  | **FN** | 0.32 ± 0.20 | 0.34 ± 0.19 | 0.28 ± 0.17 | 0.32 ± 0.18 | 0.33 ± 0.20 | 0.35 ± 0.20 |

1. pLDDT Scores: The pLDDT scores for each group represent the mean and standard deviation of the pLDDT scores for the residues within that group.
2. IUPred Scores: The IUPred scores for each group illustrate the mean and standard deviation of the residue scores within the group.TP, TN, FP and FN, prediction error analysis from each prediction models.
3. TP, TN, FP, and FN: These terms refer to the analysis of prediction errors derived from each prediction model.
4. pLDDT, IUPred, and LSTM: These models include the basic pLDDT model (residues with pLDDT < 70 are classified as “hard missing”), the IUPred model (residues with IUPred ≥ 0.5 are regarded as “hard missing”), and the advanced LSTM model trained on X-ray data provided in this study.
5. Short and Long: These terms refer to contiguous “hard missing” residues, classified as either ≤ 30 amino acid residues (short) or > 30 amino acid residues (long).

| **S5 Table. Prediction scores of “Hard missing” residues by different models in regions from SPA dataset** | | | | | | | |
| --- | --- | --- | --- | --- | --- | --- | --- |
| **Models** |  | **pLDDT** | | **IUPred** | | **LSTM** | |
| **Regions** |  | **Short** | **Long** | **Short** | **Long** | **Short** | **Long** |
| **pLDDT Scores** | **TP** | 49.47 ± 11.11 | 39.15 ± 10.62 | 51.42 ± 16.56 | 43.96 ± 17.13 | 47.55 ± 10.89 | 38.11 ± 9.76 |
|  | **TN** | 84.01 ± 18.91 | 88.49 ± 11.93 | 83.14 ± 18.99 | 87.21 ± 13.96 | 83.79 ± 18.75 | 88.13 ± 12.36 |
|  | **FP** | 58.02 ± 9.86 | 47.87 ± 11.61 | 71.93 ± 23.96 | 74.79 ± 20.60 | 53.03 ± 10.68 | 44.89 ± 11.43 |
|  | **FN** | 69.68 ± 21.39 | 80.57 ± 15.34 | 58.84 ± 18.84 | 66.27 ± 24.21 | 69.64 ± 19.16 | 78.62 ± 16.65 |
| **IUPred Scores** | **TP** | 0.40 ± 0.24 | 0.59 ± 0.24 | 0.65 ± 0.12 | 0.75 ± 0.13 | 0.41 ± 0.24 | 0.60 ± 0.24 |
|  | **TN** | 0.27 ± 0.22 | 0.22 ± 0.18 | 0.25 ± 0.21 | 0.22 ± 0.16 | 0.26 ± 0.22 | 0.22 ± 0.18 |
|  | **FP** | 0.33 ± 0.22 | 0.37 ± 0.23 | 0.59 ± 0.09 | 0.67 ± 0.11 | 0.38 ± 0.24 | 0.37 ± 0.23 |
|  | **FN** | 0.35 ± 0.23 | 0.32 ± 0.21 | 0.30 ± 0.20 | 0.31 ± 0.18 | 0.34 ± 0.22 | 0.34 ± 0.22 |

1. pLDDT Scores: The pLDDT scores for each group represent the mean and standard deviation of the pLDDT scores for the residues within that group.
2. IUPred Scores: The IUPred scores for each group illustrate the mean and standard deviation of the residue scores within the group.TP, TN, FP and FN, prediction error analysis from each prediction models.
3. TP, TN, FP, and FN: These terms refer to the analysis of prediction errors derived from each prediction model.
4. pLDDT, IUPred, and LSTM: These models include the basic pLDDT model (residues with pLDDT < 70 are classified as “hard missing”), the IUPred model (residues with IUPred ≥ 0.5 are regarded as “hard missing”), and the advanced LSTM model trained on SPA data provided in this study.
5. Short and Long: These terms refer to contiguous “hard missing” residues, classified as either ≤ 30 amino acid residues (short) or > 30 amino acid residues (long).

| **S6 Table. Prediction scores of “Hard missing” residues by different models in regions from Tomo dataset** | | | | | | | |
| --- | --- | --- | --- | --- | --- | --- | --- |
| **Models** |  | **pLDDT** | | **IUPred** | | **LSTM** | |
| **Regions** |  | **Short** | **Long** | **Short** | **Long** | **Short** | **Long** |
| **pLDDT Scores** | **TP** | 50.48 ± 11.21 | 37.52 ± 9.64 | 49.36 ± 14.44 | 38.90 ± 12.73 | 48.29 ± 10.74 | 36.69 ± 8.67 |
|  | **TN** | 81.96 ± 20.25 | 87.48 ± 12.23 | 81.21 ± 20.13 | 86.01 ± 14.50 | 81.72 ± 20.05 | 87.09 ± 12.63 |
|  | **FP** | 57.13 ± 10.79 | 45.73 ± 12.59 | 68.85 ± 24.59 | 70.61 ± 23.20 | 51.47 ± 10.74 | 41.38 ± 10.84 |
|  | **FN** | 71.98 ± 18.63 | 81.04 ± 13.75 | 61.29 ± 18.26 | 64.28 ± 24.43 | 71.12 ± 17.07 | 79.65 ± 14.81 |
| **IUPred Scores** | **TP** | 0.33 ± 0.22 | 0.59 ± 0.21 | 0.63 ± 0.11 | 0.73 ± 0.12 | 0.33 ± 0.22 | 0.60 ± 0.20 |
|  | **TN** | 0.28 ± 0.22 | 0.23 ± 0.17 | 0.26 ± 0.20 | 0.22 ± 0.16 | 0.28 ± 0.21 | 0.23 ± 0.17 |
|  | **FP** | 0.32 ± 0.20 | 0.40 ± 0.24 | 0.58 ± 0.08 | 0.66 ± 0.11 | 0.35 ± 0.20 | 0.42 ± 0.25 |
|  | **FN** | 0.26 ± 0.21 | 0.29 ± 0.17 | 0.25 ± 0.18 | 0.34 ± 0.18 | 0.26 ± 0.21 | 0.30 ± 0.18 |

1. pLDDT Scores: The pLDDT scores for each group represent the mean and standard deviation of the pLDDT scores for the residues within that group.
2. IUPred Scores: The IUPred scores for each group illustrate the mean and standard deviation of the residue scores within the group.TP, TN, FP and FN, prediction error analysis from each prediction models.
3. TP, TN, FP, and FN: These terms refer to the analysis of prediction errors derived from each prediction model.
4. pLDDT, IUPred, and LSTM: These models include the basic pLDDT model (residues with pLDDT < 70 are classified as “hard missing”), the IUPred model (residues with IUPred ≥ 0.5 are regarded as “hard missing”), and the advanced LSTM model trained on Tomo data provided in this study.
5. Short and Long: These terms refer to contiguous “hard missing” residues, classified as either ≤ 30 amino acid residues (short) or > 30 amino acid residues (long).
